# Clinical features in four patients with syphilitic outer retinopathy

**DOI:** 10.1007/s10384-025-01191-6

**Published:** 2025-04-11

**Authors:** Tomoyuki Oga, Kazuki Kuniyoshi, Akihiko Sugino, Tomoko Sato, Fukutaro Mano, Masuo Sakamoto, Chiharu Iwahashi, Koji Sugioka, Shunji Kusaka

**Affiliations:** 1 Department of Ophthalmology, Kindai University Faculty of Medicine, 377-2 Ohno-higashi, Osaka-sayama, Osaka 589-8511 Japan; 2 Department of Ophthalmology, Kindai University Nara Hospital, Ikoma, Japan

**Keywords:** Syphilis, Syphilitic outer retinopathy, AZOOR, MEWDS, Myopia

## Abstract

**Purpose:**

To present the clinical findings of patients diagnosed with syphilitic outer retinopathy.

**Study design:**

A retrospective clinical study.

**Patients and Methods:**

The study involved four Japanese men whose medical charts were reviewed and analyzed retrospectively.

**Results:**

All patients declined decimal visual acuity to 0.1, 0.2, 0.4, 0.6, and 0.8. Their fundus appearances were generally normal, except for one patient who displayed faint white dots in the upper macular region. Two patients showed dye leakage from retinal vessels during fluorescein angiography. Optical coherence tomography (OCT) results revealed abnormalities in all patients: three had a disrupted ellipsoid zone (EZ) and hyper-reflective nodules at the retinal pigment epithelium line, while one patient presented with a blurred EZ. The patients had myopia ranging from –4.5 to –7.5 diopters. They were diagnosed with syphilitic outer retinopathy based on the findings from the fundus examination, OCT, and serologic tests and were treated with oral amoxicillin, resulting in improved vision.

**Conclusions:**

The OCT findings are typical for diagnosing syphilitic outer retinopathy. Myopia was prevalent in the four patients, indicating a need for further research on refractive errors in patients with syphilitic outer retinopathy.

## Introduction

Syphilis first spread in Europe around the end of the 15th century, at the beginning of the Age of Exploration [1]. It is a sexually transmitted disease caused by *Treponema pallidum*. With the emergence of penicillin, cases of syphilis decreased substantially in the latter half of the 20th century; unfortunately, the prevalence of syphilis and ocular syphilis is now rapidly rising globally [2], including in Japan [3–6]. While the exact reason for these increases is unknown, it may have links to globalization [7] and declining AIDS-related mortality over the past two decades [8] since syphilis often occurs as a coinfection with the human immunodeficiency virus (HIV) [9].

Acquired syphilis is clinically categorized into four stages: primary, secondary, latent, and tertiary [10]. Ocular infections caused by *Treponema pallidum* (ocular syphilis) can develop at any stage and present in various forms, including conjunctivitis, episcleritis, scleritis, optic neuritis, and chorioretinitis. This encompasses conditions such as iridocyclitis, retinitis, and uveitis [11]. Chorioretinitis is the most common condition found in patients with ocular syphilis, characterized by exudates, hemorrhage, retinal and choroidal necrosis, as well as vitreous opacity [11]. Coinfection with HIV may result in unusual and rapid clinical progression [11]. Ocular syphilis stems from systemic syphilis, and its diagnosis involves both ocular and systemic assessments, along with serological tests to confirm exposure and/or active infection of *Treponema pallidum*. In 1988, de Souza and colleagues described syphilitic uveitis, which presented with a large, unilateral, yellowish placoid lesion [12]. Gass introduced the term “acute syphilitic posterior placoid chorioretinitis (ASPPC)” to characterize a unique type of posterior uveitis [13]. Gass identified ASPPC as part of the acute zonal occult outer retinopathies (AZOOR) complexes.

In 2014, Lima et al. described patients with ocular syphilis that resembled AZOOR and termed this condition “syphilitic outer retinopathy” [14]. The condition is reported in a number of papers [14–23].

We present four Japanese male patients diagnosed with syphilitic outer retinopathy. OCT revealed significant abnormalities, including a disrupted ellipsoid zone (EZ) and hyper-reflective nodules within the retinal pigment epithelium (RPE) line, whereas fundus examination appeared unremarkable.

## Patients and methods

All research protocols received approval from the Ethics Review Board of Kindai University Faculty of Medicine. The study involved four Japanese men diagnosed with syphilitic outer retinopathy at Kindai University Hospital. After obtaining written informed consent from the participants, their medical records were reviewed and analyzed retrospectively.

## Results

Table 1 summarizes the clinical findings of the four patients. Below are detailed descriptions of their clinical courses.

**Table 1:** Four patients with syphilitic outer retinopathy

Patient	Age (y) /sex	Chief complaints	Affected eye	Worst BCVA (OD/OS)	Final BCVA (OD/OS)	Refractive errors (OD/OS)	Fundus	FA	OCT	Visual field
1	41/M	Shadowed vision	OD	0.8	1.2	−7.5 D	Normal	Retinal vessel leakage	Disrupted EZ, nodules in the RPE line	Central scotoma
2	48/M	Blurred vision	OS	0.6	0.9	−5.5 D	Faint white dots	Slight retinal vessel leakage	Disrupted EZ, nodules in the RPE line	Reduced central sensitivity
3	65/M	Worsening vision, floaters	OU	0.2 0.1	0.9 1.0	−5.5D −4.5 D	Normal	(NA)	Blurred EZ	Reduced central sensitivity
4	57/M	Blurred vision	OU (OD > OS)	0.4 1.5	1.2 1.5	−5.5 D −5.0 D	Normal	(NA)	Disrupted EZ, nodules in the RPE line	Reduced central sensitivity

*y* years old, *M* male, *OD* right eye, *OS* left eye, *OU* both eyes, *BCVA* best-corrected visual acuity, *D* diopters, *FA* fluorescein fundus angiography, *NA* not available, *OCT* optical coherence tomography, *EZ* ellipsoid zone, *RPE* retinal pigment epithelium

**Fig. 1:**
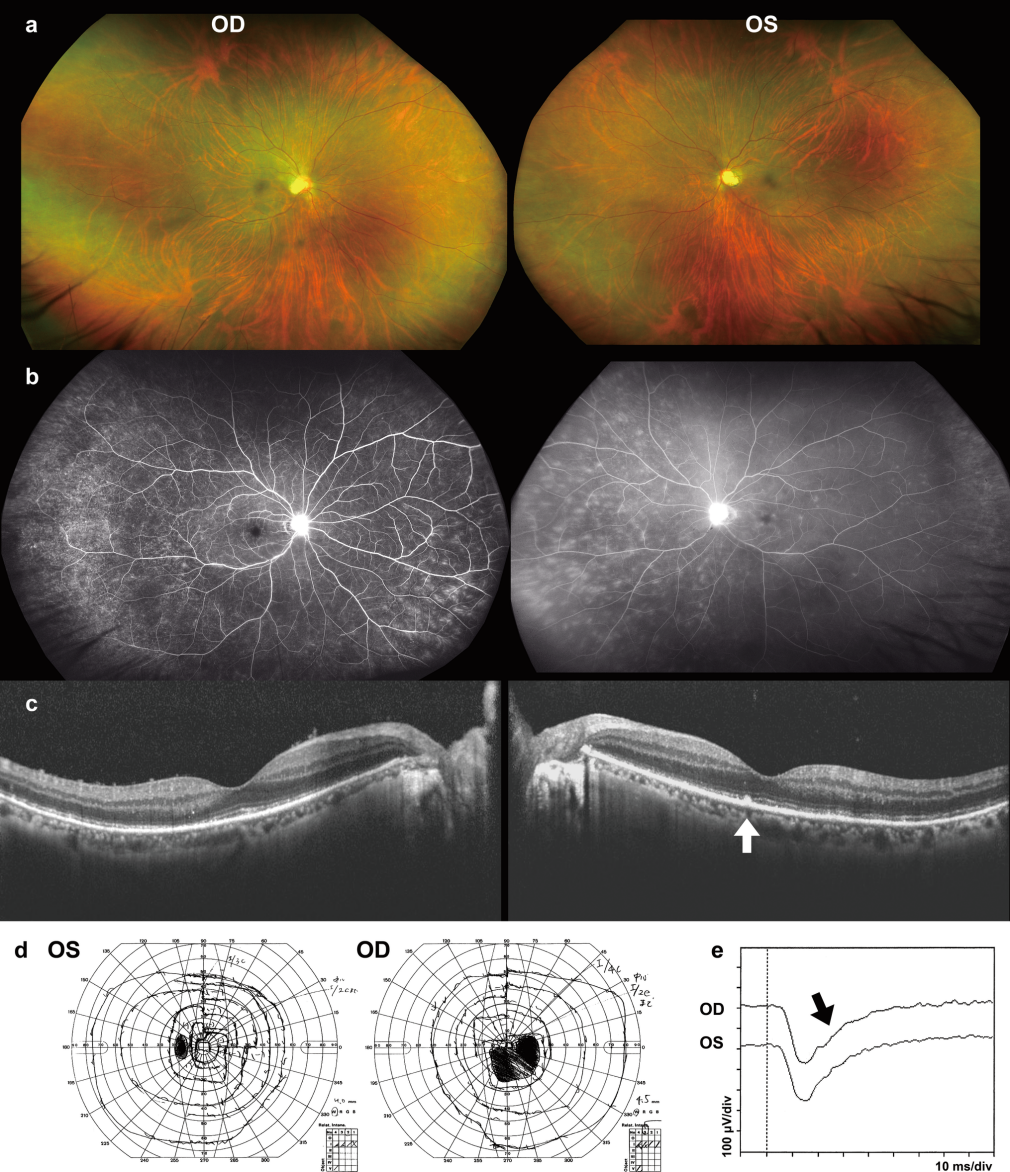
Images of fundus photographs (**a**), fluorescein fundus angiography (FA) (**b**), optical coherence tomography (OCT) (**c**), Goldmann kinetic perimetry (**d**), and flash electroretinography (ERG) (**e**) for *Patient 1.* The OCT images reveal disruption of the ellipsoid zone (EZ) in both eyes (OU), along with a hyper-refractive nodule appearing at the retinal pigment epithelium (RPE) line in the left eye (OS) (indicated by the white arrow in c). Vitreous cells are observed OU. Oscillatory potentials are extinguished in the flash ERG (marked by the black arrow in **e**)

*Patient 1* was a 41-year-old man who reported seeing “a large shadow in his right vision.” His decimal visual acuity (VA) measured 0.06 (1.0 x S-7.5 diopters (D) = C-1.0D Ax 70º) in the right eye (OD) and 0.08 (1.2 x S-6.0D = C-1.0D Ax 95º) in the left eye (OS). The corneas appeared clear, with no apparent inflammation in either eye. Fundoscopy results were unremarkable (Fig. 1a). However, OCT revealed a disrupted EZ and hyper-reflective nodules in the RPE line OS (indicated by white arrow in Fig. 1c). Additionally, both eyes contained vitreous cells (Fig. 1c).

Three months later, his best-corrected visual acuity (BCVA) OD dropped to 0.8, and he exhibited a significant scotoma OD (Fig. 1d). While fundus examinations appeared normal, and fluorescein fundus angiography (FA) revealed dye leakage from the retinal vessels, suggesting diffuse phlebitis (Fig. 1b). Full-field flash electroretinography (ERG) showed extinguished oscillatory potentials OU (black arrow, Fig. 1e).

Laboratory tests indicated a high rapid plasma reagin (RPR) titer of 156 R.U. (normal range: <1.0 R.U.) and a reactive *Treponema pallidum* hemagglutination assay (TPHA) at 1:20480 (normal range: <1:80). The anti-HIV antibody test was negative. Ultimately, he was diagnosed with syphilitic outer retinopathy. We referred him to the dermatology department at another hospital, and he began 4 weeks of oral treatment with amoxicillin.

**Fig. 2:**
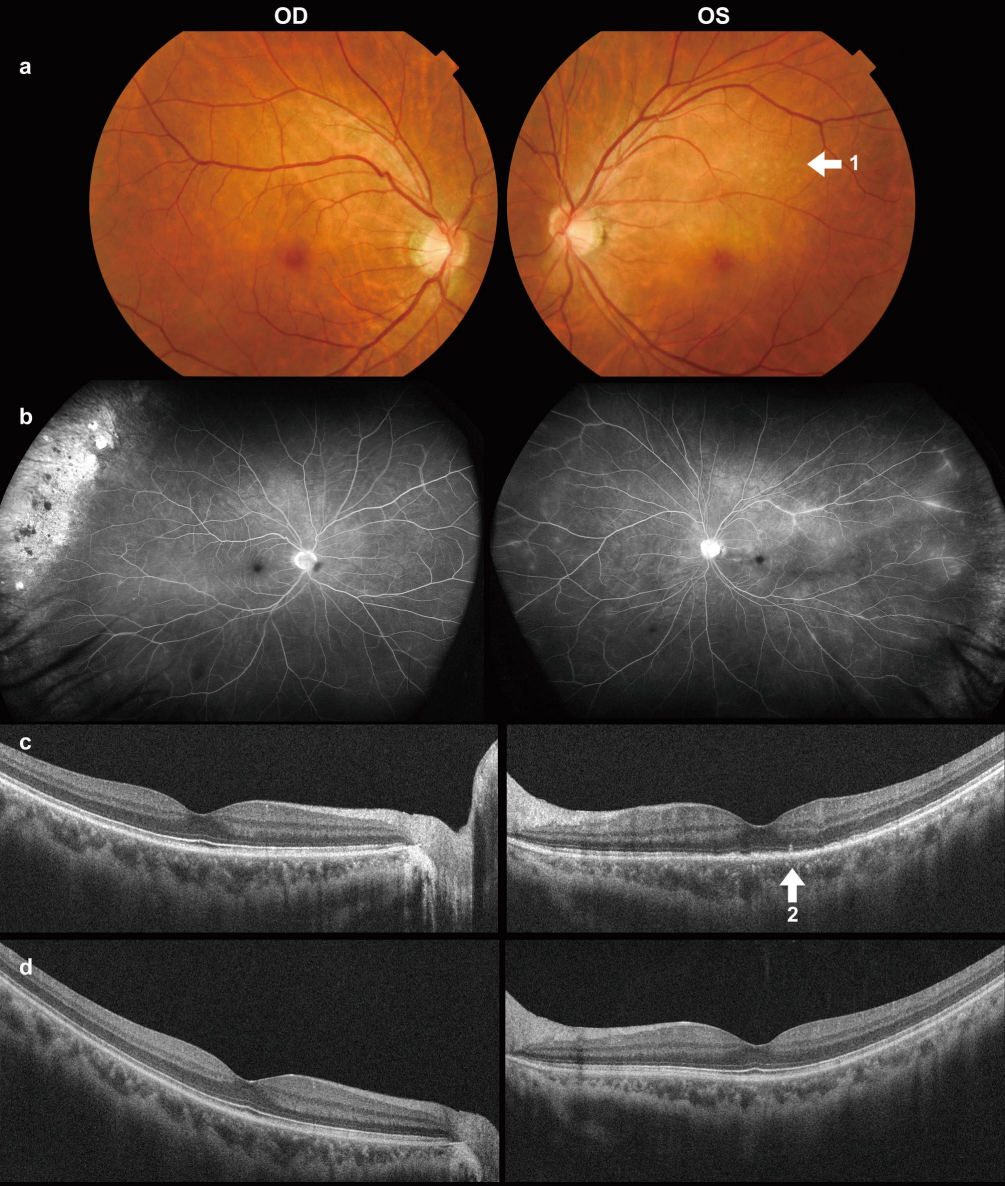
Fundus photographs (**a**), results of FA (**b**), and OCT at the initial visit (**c**), as well as OCT results at 4 months after starting treatment (**d**) for *Patient 2.* In the OCT image from the initial visit, the EZ is discontinuous, and a hyper-refractive nodule is present at the RPE line (arrow 2 in c). Four months later, the EZ was regenerated and is now continuous (**d**)

*Patient 2* was a 48-year-old Japanese man who presented with subacute decline and blurred vision OS. He had a history of diabetes mellitus. His VA measured 0.06 (1.2 x S-5.75D=C-1.0D Ax 20º) OD and 0.02 (0.6 x S-5.5D=C-0.75D Ax 120º) OS. The corneas appeared clear, and no apparent inflammation was observed. Fundus examination showed mostly unremarkable findings, except for faint white dots in the left macula’s upper area (Fig. 2a, arrow 1). FA showed little dye leakage from the retinal vessels OS (Fig. 2b). OCT revealed a disrupted EZ and a hyper-reflective nodule along the RPE line (Fig. 2c, arrow 2). Initially, the authors considered multiple evanescent white dot syndrome (MEWDS). Yet, they dismissed it after indocyanine green fundus angiography (IA) produced unremarkable findings, with no hypo-fluorescent dots present in the late phase of IA.

He was ultimately diagnosed with syphilitic outer retinopathy, indicated by a high RPR titer of 174 R.U. and a reactive TPHA result at 1:10240. The anti-HIV antibody test was negative. He commenced a 6-week treatment of oral amoxicillin. After four months, his left BCVA improved to 0.9, and the EZ was regenerated and appeared continuous on the OCT (Fig. 2d). That marked his final visit to our hospital, and clinical follow-up was interrupted.

**Fig. 3:**
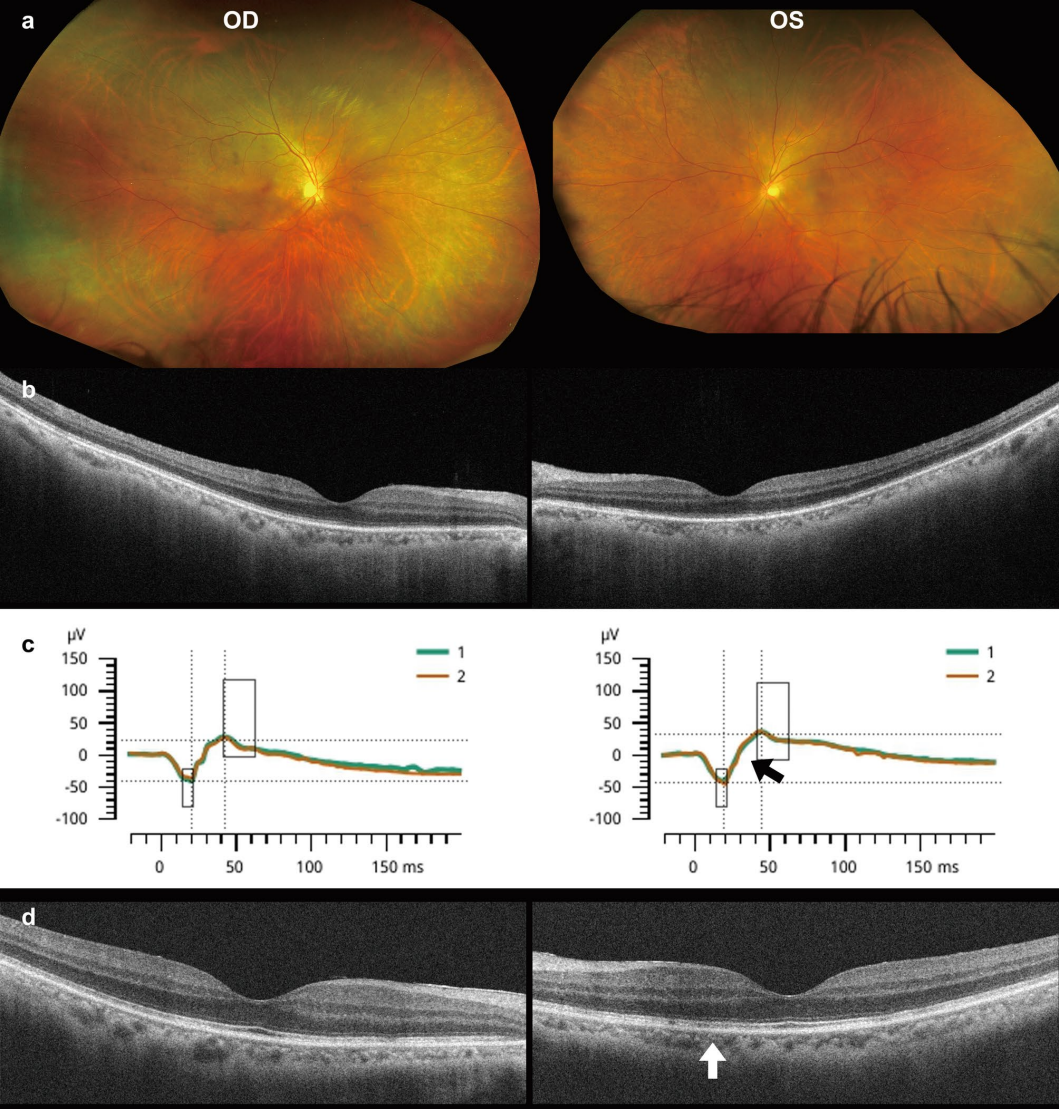
Fundus photographs (**a**), OCT (**b**), and ERG (**c**) results from the initial visit, along with the OCT image taken 4 months after starting treatment (**d**) for *Patient 3.* At the initial visit, the EZ appears blurred in the OCT image OU (**b**). Four months later, the EZ remains partially disrupted OS (indicated by the white arrow in **d**)

*Patient 3* was a 65-year-old Japanese man who presented with complaints of worsening vision and floaters OU. His VA measured 0.03 (0.2 x S-5.5D=C-0.75D Ax 20º) OD and 0.03 (0.1 x S-4.5D=C-1.0D Ax 120º) OS. The corneas were clear, and there was no apparent inflammation. Fundoscopic examination showed unremarkable findings (Fig. 3a); however, OCT showed blurred EZ OU (Fig. 3b). The flash ERG results from the left eye indicated extinguished oscillatory potentials (black arrow, Fig. 3c). He was ultimately diagnosed with syphilitic outer retinopathy due to a high RPR titer of 1100 R.U. and a reactive TPHA result of 1:20480. The anti-HIV antibody test was negative. He was treated with oral amoxicillin for 4 weeks. Four months later, his BCVA improved to 0.9 OD and 1.0 OS. However, the ellipsoid zone remained slightly discontinuous in the OCT images (white arrow, Fig. 3d). As his vision had improved he opted for reduced clinical follow-up.

**Fig. 4:**
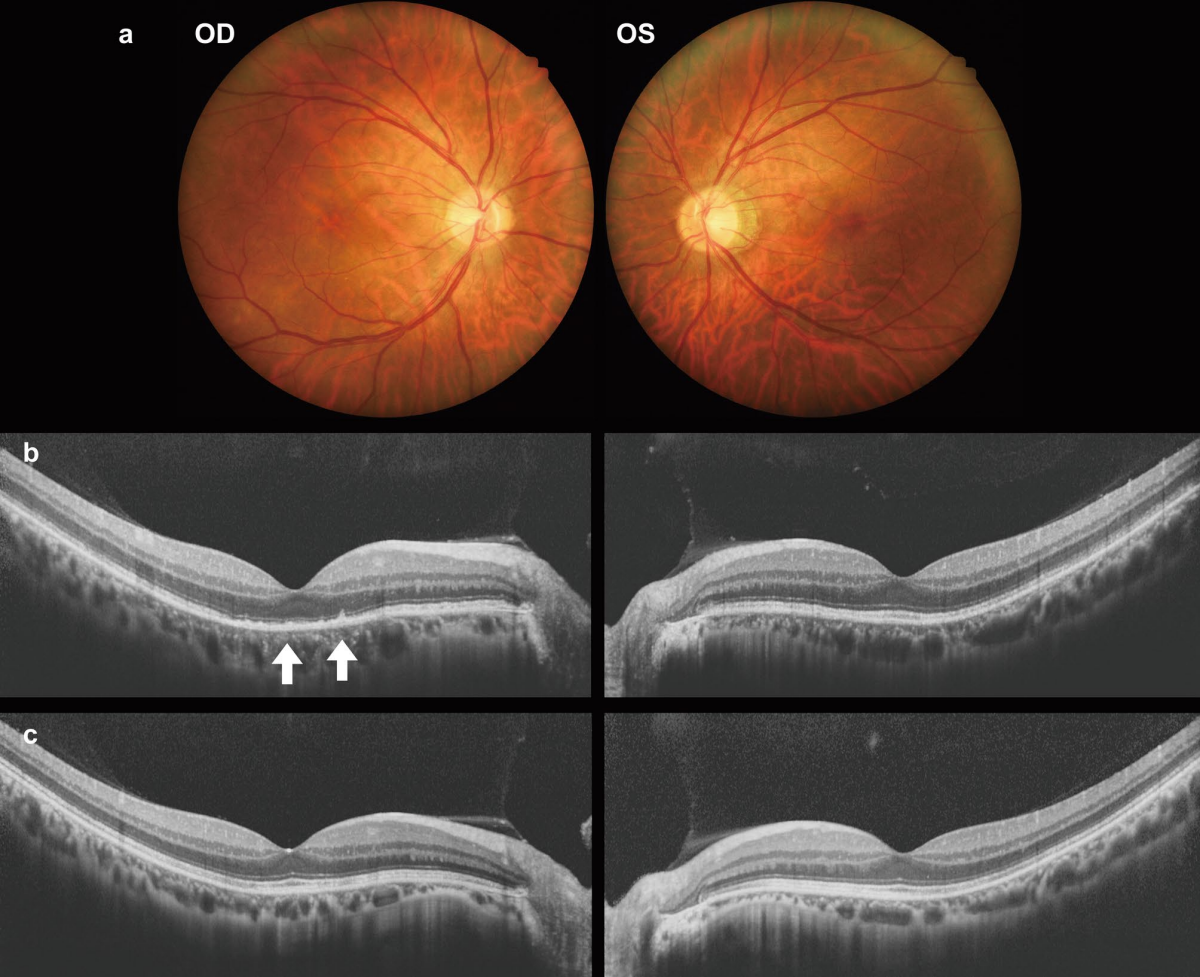
Fundus photographs (**a**) and OCT results at the initial visit (**b**) and 6 months after starting treatment (**c**) for *Patient 4.* The images show interrupted EZ and hyper-refractive nodules in the RPE line OD (marked by arrows in b) and vitreous cells OU during the initial visit (**b**). After treatment the EZ becomes continuous (**c**)

*Patient 4* was a 57-year-old Japanese man experiencing blurred vision OD. Two months prior, he had consulted an ophthalmologist and diagnosed with episcleritis. His VA was 0.04 (0.4 x S-5.5D) OD and 0.08 (1.5 x S-5.0D) OS. The corneas appeared clear, with no apparent inflammation in either eye. While funduscopy showed unremarkable results (Fig. 4a), OCT indicated a disrupted EZ and a hyper-reflective nodule located in the RPE line OD (arrows, Fig. 4b), along with vitreous cells OU. He was diagnosed with syphilitic outer retinopathy based on serological test results showing a high RPR titer of 408 R.U. and a reactive TPHA at 1:20480. Anti-HIV antibodies were not detected. He received an 8-week course of oral amoxicillin. Six months later, his decimal BCVA OD improved to 1.2, with regeneration of the EZ appearing continuous and hyper-reflective nodules disappearing on the OCT (Fig. 4c).

## Discussion

### Diagnosis and treatment of syphilitic outer retinopathy

Syphilitic outer retinopathy represents a range of syphilitic chorioretinitis, yet it often presents with either normal or only slightly abnormal fundus findings, complicating the diagnosis [14–23]. FA and OCT results were crucial in raising suspicion for syphilitic outer retinopathy in these patients. FA indicates dye leakage from retinal vessels, which points to phlebitis [14, 16, 18], and OCT shows significant abnormality in the outer retina, especially hyper-reflective nodules in the RPE line and disruption of EZ [14–19, 21]. The OCT abnormalities appear to be definitive indicators of syphilitic outer retinopathy [16–19, 21] and ASPPC [24–28]; however, these two diseases present distinct fundus appearances.

The authors believe that suspecting syphilitic retinopathy in patients with the mentioned OCT/FA abnormalities is crucial. Subsequently, serological tests lead to a final diagnosis and appropriate treatment.

Intravenous penicillin G is used to treat syphilitic outer retinopathy, similar to systemic syphilis [29]. In Japan, oral amoxicillin has been used to treat syphilis, and since 2021, intravenous penicillin G has also been available. According to the guidelines for diagnosis and treatment of systemic syphilis in Japan [30], oral amoxicillin should be continued for at least 4 weeks. Discontinuation is possible when serological test results indicate less than half of the initial RPR titer and/or below a quarter of the initial reactive TPHA value. Our patients received treatment from dermatologists following which [30], *Patients 2* and *3* exhibited incomplete recovery of visual acuity, and *Patient 3* displayed incomplete improvement in OCT findings. This indicates that the recovery of visual and OCT abnormalities may require more time than the improvement seen in serological tests, highlighting the importance of collaboration between ophthalmologists and dermatologists in treating patients with syphilitic outer retinopathy.

The outlook for syphilitic outer retinopathy is considered positive when treated appropriately [18]. While ASPPC can spontaneously resolve [31], steroids may worsen prognosis [20]. Since ocular syphilis is a manifestation of systemic syphilis, systemic treatment is essential, even if the ocular symptoms have resolved on their own.

### Retinal function in syphilitic outer retinopathy

In *Patient 1*, the flash ERG revealed diminished b-wave and extinguished oscillatory potentials (black arrow, Fig 1e). *Patient 3* exhibited normal a- and b-waves and extinguished oscillatory potentials OS (black arrow, Fig. 3c). The oscillatory potentials are believed to originate from the inner plexiform layer of the retina, including amacrine cells [32]. These ERG results indicate a dysfunction in the retina’s middle layer. As a significant reduction of the multifocal [15] or full-field ERGs [17] was reported, syphilitic outer retinopathy can functionally affect all retinal layers despite the OCT images showing abnormalities only in the outer layer of the retina.

### Syphilitic outer retinopathy, AZOOR complex, and myopia

*Patient 1* exhibited a significant scotoma and OCT irregularity despite an unremarkable fundus (Fig. 1a, d). These findings resemble those observed in AZOOR patients, as Lima et al. [14] note. *Patient 2* presented with subtle white dots in the left fundus, indicative of MEWDS; however, the diagnosis was contested due to unremarkable IA findings in *Patient 2*. Several reports [22, 23] show that syphilitic outer retinopathy could present as white dots in the fundi, mimicking MEWDS, a spectrum of the AZOOR complex. Differentiating between MEWDS and syphilitic retinopathy relies on laterality and IA findings: MEWDS typically presents unilaterally with hypo-fluorescent dots in the late IA phase, whereas syphilitic retinopathy does not exhibit these features.

Notably, all current patients exhibited myopia between –4.5 and –7.5 D (Table 1). This aligns with previous studies, where myopia was commonly observed in patients with AZOOR [33], MEWDS [34], multifocal choroiditis, and punctate inner choroidopathy [35], all of which comprise the AZOOR complex.

As previous literature has not addressed reflective errors in patients with syphilitic outer retinopathy further research involving a larger cohort is required to determine the prevalence of myopia among these patients.

A limitation of this study is the small patient population. As the clinical characteristics of syphilitic outer retinopathy are not entirely understood, it is unclear if these patients exhibit the typical features of this condition. Considering the rarity of syphilitic outer retinopathy, a multicenter study involving a larger cohort of patients would be beneficial.

In conclusion, diagnosing syphilitic outer retinopathy is challenging due to its nearly normal fundus appearance. Examinations such as OCT, FA, visual field assessments, and ERG are crucial. Pathognomonic findings for syphilitic outer retinopathy include nodular hyper-reflection in the RPE line and disruption of the EZ in OCT images.
